# Non-communicable disease prevention in Kosovo: quantitative and qualitative assessment of uptake and barriers of an intervention for healthier lifestyles in primary healthcare

**DOI:** 10.1186/s12913-022-07969-5

**Published:** 2022-05-14

**Authors:** Ariana Bytyci-Katanolli, Sonja Merten, Marek Kwiatkowski, Katrina Obas, Jana Gerold, Manfred Zahorka, Naim Jerliu, Qamile Ramadani, Nicu Fota, Nicole Probst-Hensch

**Affiliations:** 1 Department of Epidemiology and Public Health, Swiss Tropical and Public Health Institute, Allschwil, Switzerland; 2 University of Basel, Basel, Switzerland; 3 Swiss Centre for International Health, Swiss Tropical and Public Health Institute, Basel, Switzerland; 4 National Institute of Public Health Kosovo, Prishtina, Kosovo; 5 University of Prishtina, Medical Faculty, Prishtina, Kosovo; 6 Accessible Quality Healthcare Project, Prishtina, Kosovo

**Keywords:** Primary healthcare, Non-communicable diseases, Health intervention

## Abstract

**Background:**

Smoking, physical inactivity, low fruit and vegetable consumption, and obesity are common in Kosovo. Their prevention is a priority to relieve the health system of from costly non-communicable disease treatments. The Accessible Quality Healthcare project is implementing a primary healthcare intervention that entails nurse-guided motivational counselling to facilitate change in the domains of smoking, diet, alcohol consumption and physical inactivity for at-risk patients. This study quantitatively assesses the uptake of motivational counselling and the distribution of health behaviours and stages of health behaviour change of the participants according to the intervention, as well as qualitatively describes experiences and perceived benefits of motivational counselling.

**Methods:**

Study participants (*n* = 907) were recruited consecutively in 2019 from patients visiting the Main Family Medical Centres in 12 municipalities participating in the Kosovo Non-Communicable Disease Cohort study as part of the Accessible Quality Healthcare project. For the quantitative study, we used baseline and first follow-up data on smoking status, physical inactivity, obesity, fruit and vegetable as well as alcohol consumption, uptake of counselling, and stages for behavioural change. For the qualitative study, in-depth interviews were conducted with a subset of 26 cohort participants who had undergone motivational counselling.

**Results:**

Motivational counselling was obtained by only 22% of the eligible participants in the intervention municipalities. Unhealthy behaviours are high even in persons who underwent counselling (of whom 13% are smokers; 86% physically inactive; 93% with inadequate fruit and vegetable consumption; and 61% are obese); only the rate of smoking was lower in those who obtained counselling. Among smokers, over 80% were still in the pre-contemplation phase of behaviour change. More advanced stages of behaviour change were observed among the highly prevalent group of inactive persons and participants with poor dietary habits, among the 5 intervention municipalities. According to the qualitative study results, the participants who obtained motivational counselling were very satisfied with the services but requested additional services such as group physical activity sessions and specialized services for smoking cessation.

**Conclusions:**

More tailored and additional primary health care approaches in accordance with patients’ views need to be considered for the motivational counselling intervention to reach patients and efficiently facilitate lifestyle behaviour change.

**Supplementary Information:**

The online version contains supplementary material available at 10.1186/s12913-022-07969-5.

## Introduction

Adaptation of health systems to the efficient prevention and treatment of Non-Communicable Diseases (NCDs) is a priority in most all countries [1–3]. The health systems of low and middle-income countries (LMICs), which are characterized by fragmented health-care services, are particularly challenged in treating people with NCDs and multi-morbidities [4].

The burden of NCDs is attributabled substantially to behavioural risk factors such as physical inactivity, unhealthy diet, harmful use of alcohol and smoking. The behavioural risk factors are in principle cost-effective targets for NCD control [5] and are included as primary targets in the World Health Organization’s (WHO) list of “best buys” for global NCD control. Yet, in LMICs, these interventions are not sufficiently implemented. This jeopardizes LMICs from their reaching the Sustainable Development Goal to reduce premature mortality from NCDs by one-third by 2030 [2]. Considering that there is a lack of complementary services to manage risk factors for NCDs (such as smoking cessation, dietary counselling, and structured physical activity sessions), context-specific information is needed to guide LMICs in their specific NCD prevention approaches.

The Primary Health Care (PHC) setting is the most appropriate place to tackle modifiable health behaviour problems. PHC providers are most likely to see patients that present these issues before the onset of clinical disease [3, 6, 7].

There is sufficient evidence showing that healthcare providers can play a vital role in helping and motivating patients to modify NCD risk behaviours. It has been reported that even during routine primary care consultations, patients welcome advice about behaviour change, and healthcare professionals are a trusted source of health behaviour change advice [8]. In addition, a systematic review showed that in interventions, nurses were the most widely used health coaches [9].

However, behaviour change is difficult and individual choices are greatly influenced by broader social, cultural and environmental factors [10]. One of the behavioral change models named Transtheoretical model (TTM) shows that behavior change is a process and different people are in different stages of change and readiness. The results of several studies have shown the effectiveness of TTM when used, in interventions to prevent chronic conditions such as diabetes and different forms of cancers [11].

An important component of effective behavioural counselling interventions is to engage patients actively in self-management practices that are needed to change and maintain healthy behaviours [12]. Even though patients might have some knowledge about the importance of health behaviours such as healthy eating, smoking cessation, and physical activity, they may not fully understand how to convert this knowledge into an actual behaviour change [13]. This may explain in part why many individuals do not get involved in these behaviours despite convincing evidence that engaging in the group of four health behaviours (physical activity, not smoking, eating a healthy diet, and drinking alcohol in moderation) leads to a delay of 11–14 years in all-cause mortality [14].

Unhealthy behaviours are prevalent in Kosovo, a country with a comparatively low life expectancy in its regional context. Based on a recent WHO STEP wise approach to surveillance (STEPS) survey conducted among persons 25–64 years old, the prevalence of current smoking was 32% (higher in men: 42%) [15]. In a population-based survey among older people, only 14% of the study population reported regular physical activities (20% in men; 9% in women) [16]. An examination of anthropometric and physical fitness parameters in 14 to 15-year-old adolescents living in rural and urban areas, reported a high prevalence of overweight and obesity even in this young segment of the population [17].

PHC and family medicine are “points of entry” to improve this situation as pointed out by a WHO-Rapid Assessment on PHC in Kosovo [18] The Accessible Quality Healthcare Project (AQH) promotes and improves the quality of PHC in the public sector in Kosovo. Specifically, AQH implemented WHO ‘Packages of Essential Non-Communicable Disease (PEN) Protocols’ [19] which were adapted to the Kosovo context by local experts. Therefore, Health Resource Centres delivering motivational counselling were embedded into local Main Family Medical Centres (MFMC) starting with 5 pilot municipalities. This approach of delivering motivational counselling sessions by PHC nurses based on motivational interviewing approach is a way of providing one-on-one sessions to patients through empathic listening, eliciting self-motivating statements, and responding to resistance [20]. Motivational counselling based on motivational interviewing techniques for health behaviour change was shown to be effective in people with chronic conditions [21]. Qualitative evidence supports the central role of nurse-driven health promotion [10].

Implementation of WHO ‘Packages of Essential Non-Communicable Disease (PEN) Protocols’ through the AQH project started in 2018. It is timely to assess the uptake of motivational counselling sessions as well as understand patient experiences and needs during these sessions. In order to achieve safe, effective and person-centred care, it is important to pay attention and respond to patients’ feedback about their experiences of health care [22, 23]. A patient experience is defined as a reflection of what occurred during the care process is defined as patient experiences. In this way patients can provide evidence about the healthcare workers performance [24]. Furthermore, Patient Reported Outcomes (PROs) coming directly from individuals provide an understanding of how well health providers and treatments are meeting patient needs [25]. PROs help find out why a program may or may not work. In this way, PROs empower the patient to actively participate in their healthcare management [26]. Furthermore, scale-up of health interventions is facilitated by community participation [27], and patient engagement can improve the quality of care in primary care [28].

As Kosovo is lacking reliable health data, it is important to assess the health behaviour, the motivational stage for behaviour change and the patient’s view on motivational counselling in the early phase of motivational counselling. This will facilitate future assessment of the impact and it’s scaling up to improve effectiveness.

The objectives of this study, therefore, are:

1. To quantitatively assess the uptake of motivational counselling sessions, as well as the distribution of health behaviours and participants’ stages of health behaviour change according to the intervention
2. To qualitatively describe experiences and perceived benefits of PHC users towards motivational counselling.

## Methods

### Study setting

The AQH implementation project is a Swiss Agency for Development and Cooperation (SDC) project led by the Swiss Tropical and Public Health Institute (Swiss TPH). It was initiated in 2016 and has three goals: 1) to deliver quality services from PHC providers that respond better to communities’ needs, 2) to improve performance of health managers in guiding service delivery towards continuous quality improvement, and 3) to improve health literacy of the population and empower them to demand the right to quality services and better access to care.

Since May 2018, in 5 out of 12 AQH municipalities in Kosovo, specially trained nurses on ‘Motivational Interviewing’ provide one-to-one motivational counselling sessions. These counselling sessions are delivered to patients based on their needs at the Health Resource Centers. Initially there were only two trained nurses offering motivational counselling, but the AQH project is continuously training additional nurses in order to increase the capacity of human resources to benefit more patients through this intervention. The training was adapted for Kosovo according to ‘5 A’s Clinical Practice Guideline’ (Ask, Advise, Assess, Assist and Arrange), to facilitate behavior change in the domains of smoking, diet, alcohol use and physical inactivity for patients who are at risk of developing diabetes and/or hypertension, or those who have already been diagnosed. If a patient age 40 years or more seen in the MFMC is a smoker or is known to have one or more of the following conditions: hypertension, diabetes, history of hypertension and/or diabetes in the family (first-degree relatives), overweight or obesity, they are referred by the family doctor to the Health Resource Centre for nurse guided motivational counselling for behaviour changes.

This current study is part of the Kosovo Non-Communicable Disease Cohort (KOSCO) [29] funded by SDC as part of the AQH implementation project. The quantitative methodology drew data from the KOSCO study to assess the uptake of motivational counselling, and to describe health behaviours, and stages of health behaviour change after delivery of motivational counselling. The qualitative methodology was used to describe the experiences and perceived benefit of motivational counselling in a randomly selected subset of KOSCO participants consenting to the qualitative interviews.

KOSCO is a prospective longitudinal study nested within the AQH project and has been described in detail according to the STROBE (The Strengthening the Reporting of Observational Studies in Epidemiology Statement) guidelines for reporting observational studies. For the inclusion into the cohort, adults aged 40 years or older were recruited consecutively among patients receiving medical services for various reasons at Main Family Medical Centres, at one of the 12 AQH study municipalities. Persons were excluded from KOSCO participation if (1) they had a terminal illness, (2) were not able to understand or respond to pre-screening questions, (3) did not live in one of the 12 study municipalities or (4) lived abroad for more than 6 months of the year. KOSCO participants are being followed-up every 6 months by trained study nurses, alternating between a structured telephone interview and an in-person interview with clinical measurements. Ethical approvals for the study were obtained from Ethics Committee Northwest and Central Switzerland (reference number 2018–00994) on 11 December 2018 and the Kosovo Doctors Chamber (reference number 11/2019) obtained on 30 January 2019. Before any data were collected, participants were asked for their verbal and written consent.

### Study design

#### Quantitative methodology

##### Study population:

Participants from the KOSCO cohort who participated in the baseline (March–October 2019) and first follow-up (October 2019–February 2020) were included in the quantitative study.

##### Data collection:

For in-person interviews, data were collected within the MFMCs facilities. For structured telephone interviews, study nurses used private rooms. In both cases, questionnaire data were obtained on tablets with the OpenDataKit (ODK) software. After the completion of interviews, data was transferred to a secured server hosted by Swiss TPH.

##### Main predictor:

Participants were first asked about the municipality where they lived to identify those who were eligible for the PHC intervention. Interventions were available to residents of: Fushe Kosova, Vushtrri, Mitrovica, Malisheva and Gjakova. The uptake of motivational counselling sessions was determined by asking study participants from the five municipalities if they have ever obtained motivational counselling session with a nurse in the Health Resource Centre. This information, as well as data on the endpoints described below, was collected during the first follow-up of the study, administered as a structured telephone interview.

##### Main endpoints:

For assessing non-adherence to health behaviours, the behavioural modules from the WHO STEPwise approach to surveillance (STEPS) were used [30]. Physical activity was defined according to WHO criteria: engaging in ≤150 min/week moderate activity, or ≤ 75 min/week vigorous activity, or an equivalent combination of moderate- and vigorous-intensity activity throughout the week [31]. Unhealthy diet was assessed based on WHO criteria for fruit and vegetable intake (≤ 5 servings of fruits and vegetables consumed each day) [32]. Weight (kg) and height (cm) were measured in each study participant (at baseline only; prior to measurements, all equipment and tools were validated in each Main Family Medical Center) Obesity was defined as having the Body Mass Index (BMI) ≥ 30 [33]. Smoking status was operationalized as (smoker vs. non-smoker). Alcohol consumption was operationalized as consuming any amount of alcohol at least once during the past 30 days. This measure was chosen since the majority of the population in Kosovo are Muslim, and study results showed that less than 6% of the study participants reported ever to consume alcohol.

To assess stages of change, the Transtheoretical Model (TTM) was applied. It has been used in previous studies to understand a variety of behaviours including smoking cessation, weight control, diet, exercise achievement, etc. [5]. The stage of health behaviour change in each lifestyle domain (smoking habits; exercise habits; eating habits; alcohol intake) was obtained by participants’ responses to the following statements: a) Maintenance: ‘I took action more than 6 months ago to change my habits and I’m working hard to maintain this change.’ b) Action: ‘I am doing something to improve my habits.’ c) Preparation: ‘I have definite plans to improve my habits in the next month.’ d) Contemplation: ‘I am seriously intending to improve my habits in the next 6 months.’ e) Precontemplation: ‘I know I should improve my habits, but I don’t intend to’ [34].

##### Covariates:

Sociodemographic and family history factors were drawn from the baseline interview conducted between March and October 2019 and included sex, age, urban or rural residence, marital status, education, work status, ethnicity, and family history of diabetes and cardiovascular disease (CVD). Weight, height, and BMI were derived from the health examination baseline. Doctor’s diagnosis of diabetes, hypertension, and CVD were self-reported and drawn from the baseline and follow-up interviews.

##### Data analysis:

Analysis was restricted to participants with complete information on the above- listed variables. The study population was divided into 5 intervention municipalities (Fushe Kosova, Vushtrri, Mitrovica, Malisheva, and Gjakova) and 7 non-intervention municipalities (Drenas, Gracanica, Junik, Lipjan, Obiliq, Rahovec, Skenderaj). Within the group of 5 intervention municipalities, study population is further divided into those who received motivational counselling versus those who did not. Data from both the baseline and the first follow-up were pooled to compensate for the missing data on doctor’s diagnoses, motivational counselling, or smoking. If the response was “yes” either at baseline or follow-up, the response was recorded as “ever yes”, otherwise it was recorded as “never”. Obesity and family history of diabetes were only available from the baseline assessment. Totals (N) and percentages (%) for categorical data as well as means and standard deviations (SD) for continuous data, respectively, were calculated for the comparison of characteristics.

To assess differences in the distribution of unhealthy lifestyle behaviours at first follow-up (except for obesity, which is only available for baseline), all participants from the 5 intervention municipalities were compared to those of the 7 non-intervention municipalities. Furthermore, participants who received at least one motivational counselling session (as part of 5 intervention municipalities) were compared to participants from the 7 non-intervention municipalities, to assess to what extent the observed differences in lifestyle between intervention and non-intervention municipalities are potentially attributable to motivational counselling. Prevalence of unhealthy behaviours between the two groups were compared with chi-squared tests. Prevalence comparisons were a priori also stratified by the presence or absence of a self-reported diagnosis for diabetes or hypertension or cardiovascular disease.

The distribution of stages of behavioural change was compared between the intervention (stratified by having or not having obtained motivational counselling) and non-intervention municipalities for all participants, and separately for persons with and without a self-reported diagnosis of diabetes, hypertension or CVD. An additional analysis further restricted the sample to obese participants. Analysis of stages of behavioural change was restricted to persons who had declared physical inactivity and unhealthy eating at follow-up 1.

Statistical analyses were conducted using STATA V.16.1.

#### Qualitative methodology

Participants of the qualitative study module embraced participants of the KOSCO cohort having obtained at least one motivational counselling session from a trained nurse within the AQH project. The study participants were recruited through quota sampling [35]. In order to achieve equal representation from each municipality, at least 5 Albanian speaking participants from each intervention municipality were selected from the database of the KOSCO study with the aim of interviewing up to 30 participants.

##### Data collection and procedure:

In-depth interviews were conducted from July to September 2020. Participants were recruited until no additional codes, themes or insights emerged from the data, defined as data saturation [36], resulting in a total of 26 interviewed participants. A semi-structured interview guide was developed, pilot-tested and then revised. Questions addressed mainly the participants’ experiences and perceptions of the motivational counselling sessions, and changes observed in their health behaviours after these sessions. Participants were also asked about their socio-demographic profile. Due to the COVID-19 pandemic, the original data collection outlined in the protocol was amended from the previously planned one, in order to avoid participants’ risk of potential infection. Therefore, in-depth interviews were conducted by telephone instead of in-person. Interviews lasted approximately 30–40 min. In-depth telephone interviews were conducted in a private room by ABK, a female PhD student with a background in public health and fluent in Albanian. ABK did not have any relationship with study participants. The participants were informed about the reasons for conducting in-depth interviews, which were complementing the on-going cohort study, aiming to improve the healthcare services. The interviews were audio-recorded and notes were taken during in-depth interviews. After completion of the interviews they were transcribed verbatim. Verbal consent was obtained on the day of the interview. Later on, once the COVID-19 pandemic regulations allowed participants to meet ABK in person, the participant information sheet was provided and retrospective written consent could be obtained from all interviewed participants.

##### Data analysis:

Qualitative data were analysed using the Framework Method [37]. Data collection and analysis were conducted as an iterative process. Initial open coding of the original transcripts in Albanian was conducted manually by the first author (ABK), whereas the co-author (SM) reviewed the initial coding from the translated interviews. Focused codes were agreed upon, which were grouped into categories, themes and subthemes using MS Excel as a first step. (Supplementary Fig. 1: Coding Tree).

As the second step, a working analytical framework was developed based on the emerging themes, which was agreed between ABK and SM after manually coding the first ten transcripts. The remaining transcripts were indexed and in this way, final themes and sub-themes were consolidated. Reporting of qualitative research was according to consolidated criteria for reporting qualitative research (COREQ).

## Results

### Quantitative results

The current study sample consists of 907 KOSCO participants.

Table 1 summarizes the participant characteristics separately for those in non-intervention municipalities (*n* = 543) and those who did (*n* = 80) and did not (*n* = 284) obtain motivational counselling in intervention municipalities. The participants from the 5 intervention municipalities where motivational counselling is available were more likely to be urban-dwellers and Albanian speakers, compared to those from the 7 non-intervention municipalities. The distribution of other participant characteristics is not considerably different between the two municipality groups (intervention vs. non-intervention). When looking at the non-intervention municipalities, 55 and 68% of participants self-reported a diagnosis of diabetes and hypertension, respectively. One out of five participants said they had been diagnosed with a CVD. Twenty-three percent were current smokers and 55% were obese. The results confirm that motivational counselling is targeting participants according to WHO PEN protocol. Diseases and risk factors were highlighted among participants who had obtained motivational counselling and who had a higher age on average, were more likely to be women, widowed, or retired. These participants were more likely to have diabetes, hypertension, CVD, a family history of diabetes, or obesity in contrast to the participants who did not obtain motivational counselling irrespective of the municipality. All participants obtaining motivational counselling had at least one risk factor. They were however less likely to be smokers (14% in those having obtained motivational counselling as opposed to 22 and 23% respectively among those not obtaining motivational counselling in the intervention or non-intervention municipalities).

**Table 1:** Characteristics of the study population, by intervention versus non-intervention municipalities and by participants receiving versus not receiving motivational counselling in intervention municipalities

Characteristics	5 Intervention Municipalities (***n*** = 364)		7 Non-Intervention Municipalities (***n*** = 543)
	Received ≥ 1 motivational counselling n (%) 80 (22.0)	Did not receive motivational counselling n (%) 284 (78.0)	n (%) 543 (100.0%)
**Sex**			
Male	28 (35.0)	123 (43.3)	222 (40.8)
Female	52 (65.0)	161 (56.7)	321 (59.1)
**Age**			
Average	63.5 (SD = 6.9)	60.4 (SD = 9.0)	59.8 (SD = 9.4)
**Residence**			
Urban	60 (75.0)	150 (52.8)	183 (33.7)
Rural	20 (25.0)	134 (47.2)	360 (66.3)
**Marital status**			
Never married	1 (1.3)	2 (0.7)	8 (1.5)
Currently married	54 (67.5)	230 (81.0)	453 (83.4)
Separated	0 (0.0)	0 (0.0)	5 (0.9)
Divorced	0 (0.0)	4 (1.4)	4 (0.7)
Widow (er)	25 (31.2)	48 (16.9)	73 (13.4)
**Education**			
Primary school	48 (60.0)	187 (65.9)	336 (61.9)
Secondary school	30 (37.5)	83 (29.2)	166 (30.6)
College/University	2 (2.5)	14 (4.9)	41 (7.6)
**Work status**			
Working	8 (10.0)	49 (17.2)	99 (18.2)
House person	20 (25.0)	145 (51.1)	266 (49.0)
Retired or disabled	51 (63.8)	85 (29.9)	152 (28.0)
Unemployed	1 (1.2)	5 (1.8)	26 (4.8)
**Ethnicity**			
Albanian	75 (93.7)	267 (94.0)	486 (89.5)
Serbian	0 (0.0)	0 (0.0)	47 (8.6)
Roma, Ashkali, Egyptian, Other	5 (6.25)	17 (5.9)	10 (1.8)
**Doctors diagnosed diabetes**^c^			
Yes^a^	73 (91.2)	153 (53.8)	297 (54.7)
No	7 (8.7)	131 (46.1)	246 (45.3)
**Doctors diagnosed hypertension**^c^			
Yes^a^	71 (88.7)	204 (71.8)	369 (67.9)
No	9 (11.2)	80 (28.1)	174 (32.0)
**Doctors diagnosed CVD**^c^			
Yes^a^	30 (37.5)	69 (24.3)	111 (20.4)
No	50 (62.5)	215 (75.7)	432 (79.5)
**Family history of diabetes**			
Yes^b^	47 (58.7)	137 (48.2)	242 (44.5)
No	33 (41.2)	147 (51.7)	301 (55.4)
**Family history of CVD**			
Yes^b^	29 (36.2)	86 (30.2)	167 (30.7)
No	51 (63.7)	198 (69.7)	376 (69.2)
**Smoking**			
Yes^a^	11 (13.7)	63 (22.1)	125 (23.0)
No	69 (86.2)	221 (77.8)	418 (76.9)
**Obesity (BMI > =30 kg/m**^**2**^**)**			
Yes^b^	49 (61.2)	141 (49.6)	296 (54.5)
No	31 (38.7)	143 (50.3)	247 (45.5)
**Any of the above factors (diabetes, hypertension, CVD, history of diabetes, smoking, obesity)**			
Yes	80 (100)	272 (95.8)	523 (96.3)
No	0	12 (4.2)	20 (3.7)

^a^Yes - indicates that the participant has had the respective condition at either baseline or follow-up 1; No - indicates that the study participant has never had the condition

^b^only baseline information available

^c^based on self-reported diagnosis and not on clinical measurements

Table 2 displays the behavioural habits at follow-up 1. However, data on obesity were only available from baseline. With regards to smoking, participants without any cardio-metabolic condition were more likely to smoke than participants with at least one of these chronic conditions, irrespective of municipality group and motivational counselling. Participants who obtained motivational counselling were statistically significantly less likely to smoke than those from non-intervention municipalities, both overall (*p* = 0.05). With regards to physical inactivity, 45% of participants were inactive in the non-intervention municipalities compared to 65% in intervention municipalities (*p* < 0.001) and 86% in those obtaining motivational counselling (*p* < 0.001). Physical inactivity was not more prevalent in the presence of cardio-metabolic disease. With regards to unhealthy nutrition over 90% of participants consumed insufficient fruits and vegetables, without any observed differences between municipalities or intervention groups. With regards to alcohol consumption, in line with the Muslim culture, less than 6% of participants reported consuming alcohol. No differences by municipality, diagnosis, or participation in motivational counselling were observed. With regards to obesity, more than 50% of participants had a BMI ≥ 30 kg/m^2^ in the overall sample and among participants with at least one cardio-metabolic disease. Among participants without disease the percentage was around 45%. No consistent differences in the prevalence of obesity were observed between intervention and non-intervention municipalities or according to motivational counselling. In fact, equivalent to physical inactivity there was a tendency for higher obesity prevalence among those receiving motivational counselling.

**Table 2:** Distribution of unhealthy lifestyle behaviors^a^ comparing intervention versus non-intervention municipalities, all participants and restricted to participants obtaining motivational counselling in intervention municipalities, additionally stratified by presence/absence of at least one self-reported doctor’s diagnosis (diabetes, hypertension, CVD) overall and by presence/absence of at least one self-reported doctor’s diagnosis (diabetes, hypertension, CVD)

**All participants**						
	**5 Intervention Municipalities** **All Participants** ***n*** **= 364**	**7 Non-intervention Municipalities** ***n*** **= 543**		**5 Intervention Municipalities** **Participants who received ≥ 1 motivational counselling** ***n*** **= 80**	**7 Non-intervention Municipalities** ***n*** **= 543**	
	**Prevalence** **n (%)**	**Prevalence** **n (%)**	***p-*value**^**b**^	**Prevalence** **n (%)**	**Prevalence** **n (%)**	***p-*value**^**b**^
Currently smoking	68 (18.7)	119 (21.9)	0.24	10 (12.5)	119 (21.9)	0.05
Physically inactive (≤ 150 min moderate or ≤ 75 min of vigorous or combination of two)	235 (64.6)	243 (44.8)	< 0.001	69 (86.2)	243 (44.8)	< 0.001
Unhealthy eaters (≤ 5 servings of fruits and vegetables a day)	342 (94.0)	512 (94.3)	0.83	74 (92.5)	512 (94.3)	0.53
Alcohol consumer (consumed alcohol at least one day during the past 30 days)	16 (4.4)	21 (3.9)	0.69	3 (3.8)	21 (3.9)	0.96
Obese (BMI ≥ 30 kg/m^2^)	190 (52.2)	296 (54.5)	0.49	49 (61.3)	296 (54.5)	0.26
**Participants with at least one self-reported diagnosis**^c^						
	**5 Intervention Municipalities** **All Participants** ***n*** **= 311**	**7 Non-intervention Municipalities** ***n*** **= 456**		**5 Intervention Municipalities** **Participants who received ≥ 1 motivational counselling** ***n*** **= 79**	**7 Non-intervention Municipalities** ***n*** **= 456**	
	**Prevalence** **n (%)**	**Prevalence** **n (%)**	***p-*value**^**b**^	**Prevalence** **n (%)**	**Prevalence** **n (%)**	***p-*value**^**b**^
Currently smoking	54 (17.3)	96 (21.0)	0.20	9 (11.3)	96 (21.0)	0.04
Physically inactive (≤ 150 min moderate or ≤ 75 min of vigorous or combination of two)	204 (65.5)	205 (44.9)	< 0.001	68 (86.0)	205 (44.9)	< 0.001
Unhealthy eaters (≤ 5 servings of fruits and vegetables a day)	292 (93.8)	434 (95.1)	0.44	73 (92.4)	434 (95.1)	0.31
Alcohol consumer (consumed alcohol at least one day during the past 30 days)	13 (4.1)	16 (3.5)	0.63	3 (3.8)	16 (3.5)	0.90
Obese (BMI ≥ 30 kg/m^2^)	165 (53.0)	258 (56.5)	0.34	49 (62.0)	258 (56.5)	0.37
**Participants without any self-reported diagnosis**^c^						
	**5 Intervention Municipalities** **All Participants** ***n*** **= 53**	**7 Non-intervention Municipalities** ***n*** **= 87**		**5 Intervention Municipalities** **Participants who received ≥ 1 motivational counselling** ***n*** **= 1**	**7 Non-intervention Municipalities** ***n*** **= 87**	
	**Prevalence**	**Prevalence**	***p*-value**^**b**^	**Prevalence**	**Prevalence**	***p-*value**^**d**^
Currently smoking	14 (26.4)	23 (26.4)	0.99	1 (100.0)	23 (26.4)	0.27
Physically inactive (≤ 150 min moderate or ≤ 75 min of vigorous or combination of two)	31 (58.4)	38 (43.6)	0.09	1 (100.0)	38 (43.6)	0.44
Unhealthy eaters (≤ 5 servings of fruits and vegetables a day)	50 (94.3)	78 (89.6)	0.34	1 (100.0)	78 (89.6)	1.0
Alcohol consumer (consumed alcohol at least one day during the past 30 days)	3 (5.6)	5 (5.7)	0.98	1 (100.0)	5 (5.7)	0.07
Obese (BMI ≥ 30 kg/m^2^)	25 (47.1)	38 (43.6)	0.69	1 (100.0)	38 (43.6)	0.44

^a^Unhealthy lifestyle behaviors (smoking, physical inactivity, unhealthy eaters, alcohol consumption): Follow-up 1 data; obesity only available at baseline

^b^Chi-squared test

^c^Self-reported doctor’s diagnosis at either Baseline or Follow-up 1

^d^Fisher’s exact test

The stages of behavioural change were compared across municipalities and motivational counselling for the domains of smoking, physical inactivity, poor diet, and alcohol consumption (Table 3). With regards to smoking, over 80% of all participants irrespective of the municipality, motivational counselling, and disease state were still in the pre-contemplation phase. Among all participants, 88% were in precontemplation from intervention municipalities that obtained motivational counselling, 82% were from the intervention municipalities, but did not receive motivational counselling, and 95% from non-intervention municipalities were in the precontemplation phase. With regards to physical inactivity, 77% of participants in the non-intervention municipalities were in the precontemplation phase, whereas this percentage was around 15% in the intervention municipalities, irrespective of cardio-metabolic disease status. In the intervention municipalities, the percentage in the precontemplation stage did not differ between those who did or did not obtain motivational counselling. In fact, a higher percentage of participants in the group not having obtained motivational counselling reported to be in the maintenance phase (23% vs. 10%) and they were almost exclusively having at least one cardiometabolic disease. With regards to diet, a substantial difference was observed between intervention and non-intervention municipalities. While 69% of participants were in the precontemplation phase in the non-intervention municipalities, less than 10% were in the precontemplation or comtemplation phase in the intervention municipalities. But within the intervention municipalities a tendency for a more advanced stage of behaviour change was observed among those not having obtained motivational counselling. With regards to alcohol consumption, over two-thirds of the participants are in the precontemplation phase, yet numbers are too small to allow for a meaningful comparison between municipalities or motivational counselling categories.

**Table 3:** Stages of change according to lifestyle comparing intervention and non-intervention municipalities, all participants and restricted to participants obtaining motivational counselling in intervention municipalities, additionally stratified by presence/absence of at least one self-reported doctor’s diagnosis (diabetes, hypertension, CVD) overall and by presence/absence of at least one self-reported doctor’s diagnosis (diabetes, hypertension, CVD)

**All participants**												
	**Current Smokers**^a^ **(*n*** = 155)*			**Physically inactive**^a^ **(*n*** = 374)*			**Unhealthy eaters**^a^ **(*n*** = 831)*			**Alcohol consumers**^a^ **(*n*** = 34)*		
**Stage of change**	**Intervention Municipalities**		**Non-Intervention Municipalities**	**Intervention Municipalities**		**Non-Intervention Municipalities**	**Intervention Municipalities**		**Non-Intervention Municipalities**	**Intervention Municipalities**		**Non-Intervention Municipalities**
	**Yes**^c^	**No**^c^		**Yes**^c^	**No**^c^		**Yes**^c^	**No**^c^		**Yes**^c^	**No**^c^	
**Maintenance**	1 (12.5)	1 (2.6)	4 (3.7)	6 (9.5)	33(22.9)	24(11.6)	7(9.5)	112(42.8)	117(13.6)	0 (0.0)	3 (25.0)	1(5.3)
**Action**	0 (0.0)	5 (13.1)	1 (0.9)	14(22.2)	45(31.3)	21(10.5)	20(27.8)	57(21.8)	35(7.9)	1 (33.3)	2 (16.7)	5(26.3))
**Preparation**	0 (0.0)	0 (0.0)	0 (0.0)	30(47.6)	37(25.9)	3(1.5)	44(59.5)	67(25.6)	4(0.8)	0 (0.0)	0 (0.0)	0 (0.0)
**Contemplation**	0 (0.0)	1 (2.6)	1 (0.9)	2(3.2)	4(2.8)	0(0.0)	3(4.9)	3(1.1)	2(0.4)	0 (0.0)	0 (0.0)	0 (0.0)
**Precontemplation**	7 (87.5)	31(81.6)	103 (94.5)	11(7.5)	25(17.4)	159(76.8)	0(0.0)	23(8.8)	337(68.9)	2 (66.6)	7 (58.3)	13(64.4)
**Participants with at least one self-reported diagnoses**^b^												
	**Current Smokers**^a^ **(*n*** = 126)*			**Physically inactive**^a^ **(*n*** = 414)*			**Unhealthy eaters**^a^ **(*n*** = 706)*			**Alcohol consumers**^a^ **(*n*** = 26)*		
**Stage of change**	**Intervention Municipalities**		**Non-Intervention Municipalities**	**Intervention Municipalities**		**Non-Intervention Municipalities**	**Intervention Municipalities**		**Non-Intervention Municipalities**	**Intervention Municipalities**		**Non-Intervention Municipalities**
	**Yes**^c^	**No**^**c**^		**Yes**^c^	**No**^**c**^		**Yes**^c^	**No**^**c**^		**Yes**^c^	**No**^**c**^	
**Maintenance**	1 (14.2)	1 (3.2)	2 (2.3)	6 (9.6)	28 (23.1)	21 (12.1)	7 (9.5)	87 (40.6)	104 (24.8)	0 (0.0)	1 (11.1)	0 (0.0)
**Action**	0 (0.0)	4 (12.9)	1 (1.1)	14 (22.5)	38 (31.4)	17 (9.8)	19 (26.0)	48 (22.4)	32 (7.6)	1 (33.3)	2 (22.2)	4 (28.5)
**Preparation**	0 (0.0)	0 (0.0)	0 (0.0)	29 (46.7)	33 (27.2)	2 (1.1)	44 (60.2)	61 (28.5)	4 (0.9)	0 (0.0)	0 (0.0)	0 (0.0)
**Contemplation**	0 (0.0)	1 (3.2)	1 (1.1)	2 (3.2)	4 (3.1)	0 (0.0)	3 (4.1)	3 (1.4)	2 (0.4)	0 (0.0)	0 (0.0)	0 (0.0)
**Precontemplation**	6 (85.7)	25 (80.6)	84 (95.4)	11 (17.7)	18 (14.8)	133 (76.8)	0 (0.0)	15 (7.0)	277 (66.1)	2 (66.6)	6 (66.6)	10 (71.4)
**Participants without any self-reported diagnosis**^b^												
	**Current Smokers**^a^ **(*n*** **= 29)**			**Physically inactive**^a^ **(*n*** **= 58)**			**Unhealthy eaters**^a^ **(*n*** **= 125)**			**Alcohol consumers**^a^ **(*n*** **= 8)**		
**Stage of change**	**Intervention Municipalities**		**Non-Intervention Municipalities**	**Intervention Municipalities**		**Non-Intervention Municipalities**	**Intervention Municipalities**		**Non-Intervention Municipalities**	**Intervention Municipalities**		**Non-Intervention Municipalities**
	**Yes**^c^	**No**^**c**^		**Yes**^c^	**No**^**c**^		**Yes**^c^	**No**^**c**^		**Yes**^c^	**No**^**c**^	
**Maintenance**	0 (0.0)	0 (0.0)	2 (9.5)	0 (0.0)	5 (21.7)	3 (8.8)	0 (0.0)	25 (52.0)	13 (17.1)	0 (0.0)	2 (66.6)	1 (20.0)
**Action**	0 (0.0)	1 (14.2)	0 (0.0)	0 (0.0)	7 (30.4)	4 (11.7)	1(100)	9 (18.7)	3 (3.9)	0 (0.0)	0 (0.0)	1 (20.0)
**Preparation**	0 (0.0)	0 (0.0)	0 (0.0)	1 (100)	4 (17.3)	1 (2.9)	0 (0.0)	6 (12.5)	0 (0.0)	0 (0.0)	0 (0.0)	0 (0.0)
**Contemplation**	0 (0.0)	0 (0.0)	0 (0.0)	0 (0.0)	0 (0.0)	0 (0.0)	0 (0.0)	0 (0.0)	0 (0.0)	0 (0.0)	0 (0.0)	0 (0.0)
**Precontemplation**	1 (100)	6 (85.7)	19 (90.4)	0 (0.0)	7 (30.4)	26 (76.4)	0 (0.0)	8 (16.6)	60 (78.9)	0 (0.0)	1 (33.3)	3 (60.0)

^a^Analysis restricted to: current smokers; physical inactivity, unhealthy eaters; alcohol consumers at follow-up, respectively. Only analyzing participants who are within the 5 categories of stages of change (‘Relapse’ and ‘Refused’ categories are not included in the analysis). Stages of change: Follow-up 1 data

^b^Self-reported doctor’s diagnosis at either Baseline or Follow-up 1

^c^Yes - Received at least 1 motivational counselling session; No - Did not receive any motivational counselling session

Supplementary Table 1 is equivalent to the analyses in Table 3, but restricted to obese participants, motivational stages of change are presented for physical inactivity and unhealthy eating habits**.** Among obese persons with a cardio-metabolic condition, the percentage of participants in the precontemplation phase diminishes in all municipality and motivational counselling groups, yet this is not true for physical inactivity.

### Qualitative results

Table 4 shows the socio-demographic characteristics of the interviewed participants for qualitative methodology.

**Table 4:** Socio-demographic characteristics of interviewed participants (*n* = 26)

Socio-demographic characteristics	
**Municipality**	
Mitrovica	5
Vushtrri	6
Fushe Kosova	5
Gjakova	5
Malisheva	5
**Residence**	
Urban	16
Rural	10
**Age [years]**	
Mean (SD)	57 (5.9)
Range	41–66
**Sex**	
Male	14
Female	12
**Marital status**	
Married	24
Widowed	2
**Education**	
Primary school	9
High school	16
University degree	1
**Employment status**	
Unemployed	17
Employed	9
**Socio-economic status (SES)**	
Good	8
Moderate	11
Low	7

Qualitative findings are presented based on emerging themes, which are described along with their essential sub-theme as shown in Table 5 Three main themes that emerged from the interviews were the experience during motivational counselling sessions, perceived benefits towards motivational counselling sessions and identified needs for health behaviour change.

**Table 5:** Themes and Subthemes

Themes	Subthemes
Experience during motivational counselling sessions	Comfort feeling
	Quality of communication
Perceived benefits of motivational counselling sessions	Positive outlook for health
	Motivation
	Self-efficacy and change in health habits
Identified needs for health behaviour change	Additional services for quitting smoking
	Group physical activity sessions for women

#### Theme 1: experience during motivational counselling sessions

This theme refers to experiences that participants had during motivational counselling sessions with the nurse. In general, participants described positive experiences during these sessions and were satisfied with preventive services provided at the Health Resource Centre.

The emerging sub-themes of the experiences during motivational counselling sessions were related to how participants were feeling during the sessions and the quality of communication they had with the nurses.

##### Comfort feeling:

One of the emerging sub-themes was the comfortable feeling described by study participants while they were attending motivational counselling sessions. Participants also appreciated the fact that someone was speaking to them about health and that made them feel good.

*"It’s much better when someone talks to you because just reading the information is not enough. I like to speak with the nurses and that makes me feel good, I also told my friends to go there". (Woman, 54 years old).*

Participants valued the fact that the nurse was trying to help them improve their health through conversations. In addition, participants outlined that it is important to discuss with a health professional and express their health concerns.

*"When the nurse was speaking to me she was trying to see what I need and was trying to help me for my health. Sometimes what we need is to have nice conversations and speak to other people and to learn how to live better" (Man, 65 years old).*

##### Quality of communication:

Regarding communication between participants and nurses, it was described that advices were understandable; and the nurses motivated the participants to keep their hopes high for changing their existing habits.

*"When I was speaking with nurses I was understanding them, both nurses that work there are very nice. For example, I have only high school and I know what they are saying and can speak with them" (Woman, 52 years old).*

*"I like when they speak to me because they say in simple language what I can do for myself" (Woman, 49 years old).*

Some participants were very pleased that the nurses reinstated previously given advice from motivational counselling sessions, which encouraged participants to live healthier. Furthermore, it was viewed as a positive note that the nurses were listening to participants’ concerns and that nurses were interested in the participants’ health.

*"It was very nice because she listened to all my problems. This was something that I really liked and I want others to do the same, we need to have someone who can listen to us and speak with us because when you have diabetes you have many problems" (Man, 58 years old).*

#### Theme 2: perceived benefits of motivational counselling sessions

This theme is related to the perceived benefits of motivational counselling sessions that participant’s had towards their health behaviours. The emerging subthemes were: positive views that participants had towards their health after motivational counselling sessions; how they were motivated after sessions; and the types of changes they have started to do as part oftheir health habits.

##### Positive outlook for health:

Participants stated that motivational counselling sessions were helpful since they started to take better care of their health, and they had a better picture of where they stand with their health.

*"Well, I started to do what we were speaking about, like going out to walk in the nature, being careful with food….. Then I started to pay attention and not to buy fruits that have a lot of sugar and that are not good for people with diabetes. So after I spoke several times with the nurses I started to take better care of my health" (Man, 52 years old).*

Furthermore, some participants reported that there was an improvement in their blood sugar levels, lab tests and weight. The participants who were seeing improvements in their health parameters were feeling better and had a higher motivation to continue with maintaining their health behaviours.

*"I used to have 77kg when I first went to them, but after some time and by doing what we were talking, on what to eat and to start to walk, my weight is lower now, now I am 75kg. That’s why I will continue with their advices" (Woman, 60 years old).*

##### Motivation:

The participants who were not previously physically active and who were not having good eating habits experienced a positive benefit on motivational counselling sessions and their motivation was higher for changing their behaviour. Some participants reported that these sessions motivated them and they would convince themselves to try to think more positively about their health.

*"I have seen myself that I feel much better when I eat vegetables and yoghurt and lighter food with no fats. And now I have understood it myself and keep on doing it". (Woman, 60 years old).*

##### Self-efficacy and change in health habits:

The change in health habits and self-efficacy was another sub-theme, which derived from participants’ perception of motivational counselling sessions. The majority of participants described that they have started to do changes in physical activity and nutrition and were more confident to change these two habits.

*"I started walking one hour in the morning and sometimes in the afternoon. Also, before I did not eat breakfast but now when I come back from walking I eat it regularly. I know I can do these things that I spoke with the nurse” (Woman, 61 years old).*

#### Theme 3: identified needs for health behaviour change

Another derived theme was the needs and additional services that would help participants change their health behaviour.

##### Additional services for quitting smoking:

Participants that were smokers stated the lack of specialized services to quit smoking and highlighted the need to have such services. Furthermore, these participants reported that they were having a hard time to quit smoking compared to changing other health behaviours.

*"After I went there and I found out that I have diabetes, I started to drink my coffee with no sugar. I also used to forget to take the medicines, but now I take it on time. For walking, I always used to walk but now I do it more regularly. But, I smoke and I want to quit, but I can’t, I need something that will help me stop smoking. For example to have something different, which will help me to quit and not smoke anymore." (Man, 50 years old).*

##### Group physical activity sessions for women:

Some women stated that they would like to have physical activity sessions in a group with other same-gender peers. These participants also highlighted that through group sessions they would have the opportunity to have a stronger social support where they would meet new friends, and discuss their specific health conditions together.

*“We need also to have advice and physical activity in a group with other women, maybe someone doesn't like it but I want that. Maybe I can meet a new friend there, then we can learn from each other and see that there are also other women with diabetes and we can give each other courage" (Woman, 53 years old).*

Another participant noted that she is not knowledgeable about the types of exercises she can do, therefore with an organized group session a nurse can describe and show different exercises suitable for these participants.

*"I don't know what exercise to do myself and maybe to have something with a group and nurses can tell me what kind of exercises to do I want that. Because alone I am not interested and don't know how to do it"* (Woman, 49 years old).

## Discussion

To tackle unhealthy behaviours and improve motivation for health behaviour change, the AQH project developed and implemented a PHC intervention, where motivational counselling sessions are being delivered to patients by nurses in Main Family Medical Centres (MFMCs). Healthcare providers play a crucial health promotion role in older patients’ lives since patients have avery positive view about their providers [38]. This is reflected in the feelings that participants expressed about the motivational counselling sessions for behaviour change which addressed their personal concerns and provided encouragement for lifestyle changes. The quantitative results of the current study point to the fact that motivational counselling uptake needs to be improved and reach all patients in need. The qualitative results of the study provide insight on improving the future effectiveness of the motivational counselling intervention. While participants have a high willingness to change behaviour, additional services to help them quit smoking as well as organizing group physical activity sessions would be needed. The quantitative study results show that there is an insufficient utilization of the intervention by the PHC users. Of 364 individuals living in one of the five intervention municipalities all fulfilled the eligibility criteria for motivational counselling, but only 22.0% of the eligible participants’ obtained at least one motivational counselling session. This low number of utilizing the health intervention is also supported by another study which showed that fewer patients use health interventions and not at regular intervals as recommended by clinical guidelines [39].

There is descriptive, but not conclusive evidence that in the intervention municipalities, smoking is lower and fewer participants are in the precontemplation phase. With regards to fruit and vegetable consumption, considerably fewer participants in the intervention municipalities were in the precontemplation phase towards a healthier diet, yet a poor diet was still present in over 90% of participants. However, overall there was little difference between those having versus not having obtained motivational counselling and between intervention and non-intervention municipalities with regards to the distribution of unhealthy lifestyle behaviours. One of the reasons for not seeing a difference may be general barriers towards a healthier lifestyle, given the generally low household incomes in Kosovo. Another reason for not seeing a difference between these groups could be that for individuals to change their lifestyle behaviours, a longer period of time is required. This is also supported by another study which showed that changing lifestyle behaviours such as diet, smoking and exercise is difficult since it requires time, great effort as well as motivation [39]. In addition, the participants who obtained motivational counselling were more likely to have a cardio-metabolic condition, which may add to the challenge of increasing physical activity. This may explain in part the higher observed rate of maintenance of improved physical activity behaviour among those who did not obtain motivational compared to those who did obtain motivational counselling in the intervention municipalities (23% vs. 10%). It has been reported that there is a risk associated with increased physical activity such as vigorous exercise in individuals with CVD, even though exercise is beneficial for those patients. Therefore, pre-participation assessment of risk should be given to individuals who might have a higher likelihood of CVD, since CVD may be unrecognized and subclinical [40]. Furthermore, the lack of lifestyle difference between those who did versus did not actually obtain motivational counselling in the intervention municipalities could be due to the fact that nurses in the intervention municipalities were generally more alert in their everyday counselling to the issues of prevention with all patients. Preventive knowhow may also spread in the social networks and through media coverage in the intervention municipalities, given that they have a healthier average lifestyle in several domains compared to non-intervention municipalities. In regards to alcohol consumption, the overall very low prevalence of alcohol consumers in this setting does not provide a sufficient sample size for observing a change in the relation to motivational counselling for future analysis.

Regarding qualitative study results, participants that obtained motivational counselling sessions described positive experiences and perceptions towards the PHC intervention and willingness to start to change their health behaviours. This could be due to the fact that the nurses provided motivational counselling based on the ‘motivational interviewing’ approach, which differs from other approaches since it is directive and patient-centred and it focuses on what the patient thinks, wants and feels [39]. Main findings regarding health behaviours showed that participants were more motivated to start to change behaviours related to nutrition and physical activity compared to smoking. Furthermore, participants described their main needs for health behaviour change, such as services to help them quit smoking as well group physical activity sessions.

One of the main strengths of this study is that it provides insights into health behaviours among PHC users in Kosovo based on their experiences and perceptions. When interpreting the results the following study limitations need to be considered. There are three limitations in regards to the quantitative part of the study. *The first* limitation is that the analyses are descriptive and not analytic in nature. The short follow-up time does not yet allow for the observation of the impact of the intervention. Some of the study participants obtaining motivational counselling have only obtained one session which is not sufficient to infuse behaviour change in a person presenting any risk behaviour. In the absence of information on the exact date of motivational counselling obtained as result of time constraints in the interview, it is not possible to know the prevalence of unhealthy behaviours before and after motivational counselling. This is also the reason why relapse was not considered as motivational state of behavioural change, because a relapse may in fact have been a particularly strong reason for participating or not participating in motivational counselling. Continuing the assessments in the KOSCO cohort will help address some of these issues in the future. Fully adjusted modelling is foreseen in a separate paper planned after a longer follow-up time with a clearer timing of events. The current descriptive analysis of the subpopulations sets the stage for these future analyses and points to, but does not consider confounders and effect modifiers. We observed differences between those who did versus did not receive motivational counselling with a higher rate of retired and obese persons in the former demonstrating that motivational counselling is targeting in part those in most need. We also observed noticeable differences in behaviours and motivational stages between intervention and non-intervention municipalities irrespective of whether motivational counselling was obtained, which may reflect a potential spill-over effect within the intervention municipality to general counselling of all patients or to social and community networks. The s*econd* quantitative limitation entails the following: while consecutive sampling of study participants into the KOSCO cohort regardless of the reason for their doctor’s visit, aimed at improving the representativeness of the population aged 40 years and older suffering from health problems, it could at the same time have introduced selection bias as compared to an alternative approach of random sampling from the public PHC users in the respective municipalities. The* third quantitative limitation:* including only participants with complete data in the quantitative analysis lowered the sample size and may have introduced selection bias. If data completeness differed by the main endpoint and, in particular, if this incompleteness additionally differed by municipality and having or not having obtained motivational counselling.

Limitations with regards to the qualitative methodology include *first*, that in-depth interviews were conducted through telephone instead of in-person due to the coronavirus pandemic. Therefore, during telephone interviews body language and other non-verbal cues could not be observed from study participants. In this way, there could be some potential loss of contextual data, and potential probing from in-depth interviews could have been missed. On the other hand, evidence shows that telephone interviews are a good medium for data collection [41]. *Second,* participants were selected from five intervention municipalities through quota sampling, which is a non-random sampling strategy. Therefore, our sample for qualitative methodology was less representative and the perspective on generalizability could be limited. While an overall sample size of 30 participants for the qualitative study is reasonable and in line with the available funds, a higher sample size would have allowed to better represent the 5 intervention municipalities.

### Suggestions to improve services

The results of the quantitative study describe early differences between intervention and non-intervention municipalities and between those who did versus did not obtain motivational counselling to guide future analyses on the longer-term impact of motivational counselling. The quantitative results demonstrate that there are still potential biases whom the motivational counselling should reach and actually does reach. The results of the qualitative study contribute to strengthening the PHC intervention based on patients’ views and identified potential barriers for its impact.

To improve the health behaviour of PHC users, and by taking into consideration their experiences and needs towards this intervention the following tailored approaches are suggested: a) strengthened referral mechanism within PHCs from family doctors to nurses b) specialized services for smoking cessation and c) delivery of group physical activity sessions for PHC users.

#### Strengthen the referral mechanism within the facility

Our quantitative study results show that from five intervention municipalities only 22.0% obtained at least one motivational counselling session. Even though the uptake of motivational counselling sessions was low, qualitative findings show that participants who attended the motivational counselling sessions mainly reported positive experiences towards the sessions and were more motivated to start to change their health behaviours. Therefore, it is recommended to increase the uptake of motivational counselling sessions offered by MFMCs. In order to increase the flow of patients to receive preventive services, the referral mechanism within the facility from the family doctor to the nurse needs to be strengthened. Previous research on physical activity counselling in primary healthcare suggests an interdisciplinary model where primary care physicians refer their patients to allied health professionals for physical activity behaviour change [42].

#### Specialized services for smoking cessation

Quantitative as well as qualitative findings show that participants that are currently smoking require additional approaches for quitting smoking such as professional support from nurses or the primary healthcare system. One of the approaches would be to offer specialized services within PHC where nurses receive additional training on smoking cessation and integrate this additional service into motivational counselling sessions. Evidence shows that patients who obtained an intervention led by a nurse had a higher smoking cessation rate compared to those who obtained usual care [43]. In addition, behavioural therapy and smoking cessation aids are needed for patients that have difficulty quitting smoking [44].

#### Group physical activity sessions for PHC users

Based on qualitative findings it was evident that besides motivational counselling sessions, PHC users identified the need to have physical activity sessions in a group. The quantitative findings showed that there was low adherence to WHO recommendations for physical activity. Therefore, organizing structured group physical activity sessions for PHC users would increase adherence to WHO physical activity recommendations. Furthermore, study participants noted that group physical activity sessions would enable them to have more social support and create new friendships. Evidence on the relationship between social support and physical activity in older adults showed that people with greater social support were more likely to do leisure-time physical activity. Therefore, interventions for older adults should take into consideration the promotion of the social benefits of physical activity participation [45].

## Conclusion

The results of this study show insufficient utilization of the PHC intervention, but the participants that obtained this intervention perceived it to have a positive benefit on their lifestyle behaviours. Tailored PHC approaches in accordance with patients’ views will need to be considered to scale-up the PHC intervention and thereby facilitate lifestyle behaviour change in patients with NCDs.

## Supplementary Information

**Additional file 1: Figure 1.** Coding Tree.

**Additional file 2: Table S1.** Stages of change for physical inactivity and unhealthy eaters comparing intervention and non-intervention municipalities among obese participants, overall and by presence/absence of at least one self-reported doctor’s diagnosis (diabetes, hypertension, CVD).

## Data Availability

The datasets generated and/or analysed during the current study are not publicly available since the datasets contain medical information of study participants, but are available from the corresponding author on reasonable request.

## Abbreviations

AQH: Accessible Quality Healthcare
BMI: Body Mass Index
CVD: Cardiovascular disease
KOSCO: Kosovo Non-Communicable Disease Cohort
MFMC: Main Family Medical Centre
NCDs: Non-communicable Diseases
ODK: Open Data Kit
PHC: Primary Health Care
PRO: Patient Reported Outcome
SDC: Swiss Agency for Development and Cooperation
SES: Socio-economic status
Swiss TPH: Swiss Tropical and Public Health Institute
TTM: Transtheoretical Model
WHO: World Health Organization
WHO PEN Protocol: WHO Package of Essential Non-Communicable Disease Protocol
WHO STEPS: WHO STEPwise approach to surveillance
