# Supplementary material for: Non-communicable disease prevention in Kosovo: quantitative and qualitative assessment of uptake and barriers of an intervention for healthier lifestyles in primary healthcare
Source: BMC Health Serv Res. 2022 May 14;22:647. doi: 10.1186/s12913-022-07969-5 (PMC9107010; doi:10.1186/s12913-022-07969-5)
Supplement: Supplementary file 1 — Additional file 1: Figure 1. Coding Tree. [file 12913_2022_7969_MOESM1_ESM.docx]

Improvement in clinical measurements

Doing better

Take better care of health

**Coding Tree**

Start to change

More careful in eating habits

Positive thinking

Self-efficacy and change in health habits

**Perceived benefits of motivational counselling sessions**

Positive outlook for health

Motivation

Higher hope

Higher self-confidence

Self-encouragement

Helpful for my health

Figure 1: Experiences and perceived benefits of motivational counselling

- Unmet needs for health behavior change
- Interpersonal experiences during counselling sessions
- Perceived benefits of motivational counselling sessions

**Experiences and perceived benefits of motivational counselling**

Interested in my health

Feeling good

Nice environment

Welcomed

Comfortable

Listened to my problems

Simple language

Understandable

Quality of communication

Comfort feeling

Open conversations

Speak slowly

Kind words

**Interpersonal experiences during counselling sessions**

Something to help us quit

Don’t know how to quit

Additional services for quitting smoking

Need more time to quit

**Unmet needs for health behaviour change**

Exercise together

Group physical activity sessions

Exercise and meet people
