# Supplementary material for: Non-communicable disease prevention in Kosovo: quantitative and qualitative assessment of uptake and barriers of an intervention for healthier lifestyles in primary healthcare
Source: BMC Health Serv Res. 2022 May 14;22:647. doi: 10.1186/s12913-022-07969-5 (PMC9107010; doi:10.1186/s12913-022-07969-5)
Supplement: Supplementary file 2 — Additional file 2: Table S1. Stages of change for physical inactivity and unhealthy eaters comparing intervention and non-intervention municipalities among obese participants, overall and by presence/absence of at least one self-reported doctor’s diagnosis (diabetes, hypertension, CVD). [file 12913_2022_7969_MOESM2_ESM.docx]

**Supplementary Table 1:** Stages of change for physical inactivity and unhealthy eaters comparing intervention and non-intervention municipalities among obese participants, overall and by presence/absence of at least one self-reported doctor’s diagnosis (diabetes, hypertension, CVD)

| **All participants** | | | | | | |
| --- | --- | --- | --- | --- | --- | --- |
|  | **Physically inactive**^1^  **(n=228)** | | | **Unhealthy eaters**^1^  **(n=442)** | | |
| **Stage of change** | **Intervention Municipalities** | | **Non-intervention Municipalities** | **Intervention Municipalities** | | **Non-Intervention Municipalities** |
|  | **Yes**^3^ | **No**^3^ |  | **Yes**^3^ | **No**^3^ |  |
| **Maintenance** | 3 (8.1) | 14 (19.2) | 16 (13.6) | 2 (4.5) | 58 (45.0) | 77 (28.5) |
| **Action** | 9 (24.3) | 22 (30.1) | 10 (8.5) | 11 (25.0) | 28 (21.7) | 19 (7.9) |
| **Preparation** | 15 (40.5) | 16 (21.9) | 2 (1.7) | 28 (63.6) | 30 (23.3) | 2 (0.7) |
| **Contemplation** | 1 (2.7) | 2 (2.7) | 0 (0.0) | 3 (6.8) | 2 (1.6) | 0 (0.0) |
| **Precontemplation** | 9 (24.3) | 19 (26.8) | 90 (76.3) | 0 (0) | 11 (8.5) | 171 (63.6) |
| ^1^ **Analysis restricted to**: obese participants (BMI≥30), based on baseline data; restricted to physical inactivity and poor nutrition, respectively. Only analyzing participants who are within the 5 categories of stages of change (‘Relapse’ and ‘Refused’ categories are not included in the analysis);  Stages of change: follow-up 1 data  ^3^ Yes - Received at least 1 motivational counselling session; No - Did not receive any motivational counselling session | | | | | | |

| **Participants with at least one self-reported diagnoses**^2^ | | | | | | |
| --- | --- | --- | --- | --- | --- | --- |
|  | **Physically inactive**^1^  **(n=201)** | | | **Unhealthy eaters**^1^  **(n=388)** | | |
| **Stage of change** | **Intervention Municipalities** | | **Non-intervention Municipalities** | **Intervention Municipalities** | | **Non-Intervention Municipalities** |
|  | **Yes**^3^ | **No**^3^ |  | **Yes**^3^ | **No**^3^ |  |
| **Maintenance** | 3 (8.1) | 13 (21.3) | 14 (13.5) | 2 (4.5) | 50 (46.7) | 68 (28.6) |
| **Action** | 9 (24.3) | 19 (31.1) | 8 (7.7) | 11 (25.0) | 22 (20.5) | 17 (7.1) |
| **Preparation** | 15 (40.5) | 14 (22.9) | 1 (0.9) | 28 (63.6) | 27 (25.2) | 2 (0.8) |
| **Contemplation** | 1 (2.7) | 2 (3.2) | 0 (0.0) | 3 (6.8) | 2 (1.8) | 0 (0.0) |
| **Precontemplation** | 9 (24.3) | 13 (21.3) | 80 (77.6) | 0 (0) | 6 (5.6) | 150 (63.2) |
| ^1^**Analysis restricted to**: obese participants (BMI≥30), based on baseline data; restricted to physical inactivity and poor nutrition, respectively. Only analyzing participants who are within the 5 categories of stages of change (‘Relapse’ and ‘Refused’ categories are not included in the analysis).  Stages of change: follow-up 1 data  ^2^ Self-reported doctor’s diagnosis at either Baseline or Follow-up 1  ^3^ Yes - Received at least 1 motivational counselling session; No - Did not receive any motivational counselling session | | | | | | |

| **Participants without any self-reported diagnoses**^2^ | | | | | | |
| --- | --- | --- | --- | --- | --- | --- |
|  | **Physically inactive**^1^  **(n=27)** | | | **Unhealthy eaters**^1^  **(n=54)** | | |
| **Stage of change** | **Intervention Municipalities** | | **Non-intervention Municipalities** | **Intervention Municipalities** | | **Non-Intervention Municipalities** |
|  | **Yes**^3^ | **No**^3^ |  | **Yes**^3^ | **No**^3^ |  |
| **Maintenance** | 0 (0.0) | 1 (8.3) | 2 (13.5) | 0 (0.0) | 8 (36.3) | 9 (28.1) |
| **Action** | 0 (0.0) | 3 (25.0) | 2 (13.3) | 0 (0.0) | 6 (27.2) | 2 (6.2) |
| **Preparation** | 0 (0.0) | 2 (16.6) | 1 (6.6) | 0 (0.0) | 3 (13.6) | 21 (65.6) |
| **Contemplation** | 0 (0.0) | 0 (0.0) | 0 (0) | 0 (0.0) | 0 (0.0) | 0 (0) |
| **Precontemplation** | 0 (0.0) | 6 (50.0) | 10 (66.6) | 0 (0.0) | 5 (22.7) | 0 (0) |
| ^1^**Analysis restricted to**: obese participants (BMI≥30), based on baseline data; restricted to physical inactivity and poor nutrition, respectively; based on baseline data. Only analyzing participants who are within the 5 categories of stages of change (‘Relapse’ and ‘Refused’ categories are not included in the analysis). Stages of change: follow-up 1 data  ^2^ Self-reported doctor’s diagnosis at either Baseline or Follow-up 1  ^3^ Yes - Received at least 1 motivational counselling session; No - Did not receive any motivational counselling session | | | | | | |
